# Treatment of Erysipelas

**Published:** 1856-02

**Authors:** 


*Treatment of Erysipelas.*

M. VELPEAU gives the results of his treatment of 1000 cases of Erysipelas. He places the greatest reliance in *iron.* He employs the *proto-sulphate of iron* in solution, about twelve grains to the ounce of water—or as an ointment, eight parts to thirty of lard. In forty cases, in which this was exclusively used, the erysipelas yielded in from twenty-four to forty-eight hours. The ointment is more easily employed to some parts than the lotion, but is somewhat less efficacious. It should be applied about three times a day. The lotion should be applied by soft compresses or cloths kept constantly moist.—*Bull. de Therap.*

